# Mr. Kirby's Additional Observations on Severe Forms of Hæmorrhoidal Excrescences; with the History of an Extirpation of the Parotid Gland

**Published:** 1826-04

**Authors:** 


*Additional Observations on the Treatment of certain Severe Forms of Hæmorrhoidal Excrescence, illustrated by Cases; with the History of a Case in which an Enlarged Parotid Gland was successfully removed.* By John Kirby, A.B. lately President of the Royal College of Surgeons in Ireland, &c. 8vo. pp. 145. Dublin, 1825.

Well might the illustrious Pott aver that, "it is certainly a disease, which whoever labours under must lead a miserable existence." Hæmorrhoidal excrescences entail a great many evils, not to say dangers, on the unfortunate sufferer, in consequence of the daily protrusion and stretching of the parts, and of their vicinity to the urinary and spermatic organs, where they occasion much sympathetic disturbance. Every attempt, therefore, to remedy such malady is deserving of the serious attention of the surgical profession. In order that our readers may reap as much benefit as possible from Mr. Kirby's remarks, we shall take a rapid survey of his first pamphlet on this subject, published in the year 1817.

The removal of hæmorrhoidal excrescences by the knife is by no means so rare an operation in this kingdom as Mr. Kirby imagines; but it may be less frequently performed than it should be. The disease is one, which, from personal knowledge, we can say, renders existence miserable; and consequently every attempt towards the relief of it must be proportionably valuable. The few cases from which is constructed the present pamphlet, are as follow.

*Case* 1. Mr. Kirby once witnessed the application of a ligature to a severe hæmorrhoidal excrescence in a female about 50 years of age, who was emaciated, and exhausted by pain and hæmorrhage. The anus readily admitted the two first fingers. The integuments of its border hung like a circular tube between the nates, to the extent of two inches, assuming the appearance of a prolapsus ani, excepting in its colour and the disposition of its rugæ. Whenever she made an effort, as at stool, a number of red, firm, circular tumours descended, and produced those painful consequences which it is unnecessary to describe. These tumours were severally included in ligatures. Peritoneal inflammation; convulsions; suppression of urine came on, and nearly proved fatal. In another case, the application of a ligature was followed by tetanus and death.

*Case* 2. Mr. P. ætat. 45, had a projection of integuments at the margin of the anus, of an inch and a half, terminating in a number of soft, livid, pile-like tumours. The prolapsus was great after each stool. His face was indicative of his sufferings. Mr. K. passed a large curved needle, carrying a strong ligature, through the entire circle of the pendulous projection. Directing the patient to force gently downwards, while Mr. K. drew the parts firmly towards him, they were removed by a single stroke of the scalpel. Several veins, which lay on the surface of the tumour, were divided, but quickly ceased to bleed. Two small arteries spouted, but were soon suppressed by the ends of the fingers, and a sponge supported by the T bandage. On the third day he dressed himself, and went to his usual occupations.

*Case* 3. Hayden, 40 years of age, tall, thin, and ghastly. Anus surrounded by a flaccid circular projection covered by the common integuments externally; lined internally by a vascular tunic. The finger introduced into the anus found the intestine every where occupied by soft, round, elastic tumours, nearly painless. At stool a protrusion always took place, accompanied by acute pain and hæmorrhage. Hæmorrhage followed the operation from a small artery, which, however, was secured. He perfectly recovered.

*Case* 4. B———, Esq. ætat. 40, a free liver, of sedentary habits, and subject to internal piles for many years. Has now a *fistula in ano*, with discharge. The integuments at the margin of the anus appear elongated to the extent of an inch and better; they are tender and excoriated. The fistula was first operated on, and then the pendulous border of the anus was removed, as in the other cases. Nothing troublesome ensued.

*Case* 5. A young gentleman, 21 years of age, had been subject to piles for twelve months, accompanied by considerable distress. Every time he went to stool a bunch of tumours descended, and required moderate pressure to effect their return, with the horizontal position. The integuments, at the margin of the anus, projected a little and were soft and flabby. The pendulous portions were removed, and the gentleman quite recovered.

The remainder of Mr. K's. work is formed of quotations from, and observations on, the writings of Pott, Hey, Abernethy, &c. with several extracts in French, from a work on Hæmorrhoids by Larroque. Nothing is said on the internal treatment of piles themselves, nor on the connexion of their phenomena with certain states of the constitution; though we think our author might have more usefully filled the remainder of the work with these subjects, than with the disquisitions above mentioned.

We now come to the further results of eight years' additional experience. Within that period Mr. K. avers that several individuals have proved the value of the means he previously proposed for their relief, after surgical skill had in vain exhausted every effort for that purpose. The operation has also been performed by others of his brethren, and received their sanction. We proceed to exhibit sketches of the cases brought forward in this little volume.

*Case* 6. Mrs. ———, about 40 years of age, had enjoyed good health up to the accession of the present disease, whose severity increased rapidly, and at last put it out of her power to go into society, being compelled to keep in bed the greater part of her time. The bowels always required medicine, and the operation was most painful for some hours, attended with prolapsus of the rectum, &c. She had been a sufferer thus for one year, and had become greatly emaciated by pain and hæmorrhage. The anus was relaxed, and the integuments at its margin had grown into flaccid folds, which encircled numerous livid piles, some of them ulcerated on the surface, tense, and painful to the touch. The rectum, within the sphincter, was free from hæmorrhoids. All the hæmorrhoidal parts were removed, as in former cases, and the hæmorrhage was trifling. The dressings were simple—the bowels were daily opened by sulphate of magnesia—the parts healed rapidly, but with a tendency to contraction, which was obviated by the bougie. This operation took place eight years ago, and Mr. K. on enquiry at the present time, found that the patient was in the enjoyment of excellent health.

*Case* 7. Lieutenant B———, 68th Regiment, aged 24, consulted our author in the year 1818, his disease having then been of several years' continuance. The anus was so relaxed as easily to admit the three fingers; the integuments of its margin little elevated into tumours; but when he made an effort at stool, a considerable protrusion took place, and many internal piles were brought into view. He suffered much from hæmorrhage, causing paleness and emaciation, with irritability, and hypochondriacal depression. He had been incapacitated for duty during the preceding six weeks, and was making arrangements for quitting the service.

Dr. Reed of the regiment passed the needle and ligature, in the manner formerly recommended by our author, and the excision was tedious, in consequence of the patient's restlessness; but was at length accomplished satisfactorily, and without any considerable hæmorrhage succeeding. Under the usual local treatment the parts rapidly granulated, and in three weeks this officer returned to his duty and accompanied his regiment to America in good health.

*Case* 8. Mr. Bellingham had been attacked, in the year 1809, with hæmorrhoids, and, in the year 1816, the disease had increased so much that, at every stool, a great number of tumours came down, resembling a bunch of grapes. After each evacuation he was obliged to go through the painful process of forcing the tumours back again, and then lying for some hours in a horizontal posture to prevent their extrusion. At length the complaint became so bad that Mr. B. was confined altogether, being greatly exhausted by pain and loss of blood. In this state, Mr. K. was taken to the patient by Mr. Macnamara, of Dublin, and recommended an operation as the only effectual means of relief. After much hesitation, the patient consented, and the mass was removed in the usual way, in the presence of Mr. Carmichael and Mr. Daniel. Dossils of lint dipped in warm oil, stupes and opium, procured freedom from pain for the first three days. To prevent adhesion, the surgeon's finger was passed into the rectum each morning, and the bowels were kept soluble by saline aperients. Before the end of three weeks he was able to move about, and was soon quite well. These circumstances are substantiated by Mr. Bellingham's letter, although some degree of constriction afterwards took place, with some purulent discharge, but unattended by any pain or inconvenience.

Nineteen cases are detailed in this volume by Mr. Kirby, but we conceive it to be unnecessary to adduce more examples than those which we have given above. We shall dwell a few minutes on the "observations" with which this part of the work concludes.

Our author observes, that the dread of hæmorrhage seems to be the principal source of opposition to a more general introduction of the knife, in preference to the ligature, as a mean of removing hæmorrhoidal excrescences. This fear, he thinks, is neither warranted by any thing which he finds written on the subject, nor by any occurrences which have taken place in his own practice or the practice of others. In only one instance has he seen such a hæmorrhage as would awaken the anxiety of a surgeon, and in that case, the bleeding was quickly checked on his reaching the patient. In the majority of cases there was less loss of blood than was necessary to obviate the spasm of the parts and the painful twitches, which not uncommonly take place after the operation. Mr. K. is convinced, from repeated examinations, that "no direct or large communication exists between the veins connected in the operation and the tributary trunks of the vena portarum."

"On reference to the preceding cases it will be perceived, I confine the operation at first to such parts as are situated below the sphincter muscle. These I freely remove by a scooping motion of the knife, on the one side carried through the integuments at the base of the hæmorrhoidal mass, and at the other, through or a little above the sulcus, which marks the union of the mucous membrane and of the integuments. This depression lies within the confines of the anus, and requires the patient to force down, that it may be brought freely into view; and after the operation it retires within the membrane of the sphincter, nearly the entire wound disappearing. By the spasmodic contraction which takes place, the surfaces of the wound are forced into close apposition, and by the pressure of the muscle all chance of more than oozing hæmorrhage is prevented.—Should a surgeon divide the mucous membrane at an unnecessary distance from the sulcus, the retraction will be apt to carry away a part of the wound above the embrace of the sphincter. Then, certainly, hæmorrhage may be more likely to occur without the knowledge of either the patient or the attendant. But this accident will not happen in the hands of any person who understands the principle of the operation, and the object it is designed to accomplish." 117.

It has been remarked by our author, that the surface of the wound quickly retires within the sphincter, and the same action draws within the gut the remainder of the hæmorrhoidal tumours. In many instances these never again descend; but in some cases they come down within the first few days, and greatly aggravate the sufferings of the patient, as we can testify from personal feeling. Under such circumstances they cannot be replaced. Every attempt at such reduction is both fruitless and agonising, and should not be made. In these cases, the tumours composing the protrusion, inflame, become exquisitely sensible, and either slough away, or lose their sensibility, and shrink up into a fleshy kind of excrescence which gives no farther trouble; or, if inconvenient, may be easily removed with very little pain. This termination, though painful, is, upon the whole, highly desirable in severe hæmorrhoidal affections. It gives a certain and permanent security against returns of the complaint.

The tendency to contraction of the anus is generally attributable to inattention at the beginning, and may be easily obviated by the bougie.

"That hæmorrhoids, in their greatest simplicity of form, constitute a disease, I imagine no one will deny. That in particular constitutions they exert a salutary influence, is an opinion, against the validity of which, few will be so rash as to contend. But who that listens to the lessons of experience, or that gives due consideration to the facts which come under his own observation, can subscribe to the position that they are always dependant on constitutional derangement. In many cases they are unquestionably a local disease; in many others, although they exist with disorder of the constitution, and perhaps with organic changes amongst the viscera, still they are oftentimes neither direct causes nor effects, notwithstanding the manner in which they appear to influence the prevailing symptoms of disease. In a business which is so plain to experienced practitioners, these observations may be deemed unnecessary. But let it be remembered, I contend against a common prejudice, to which many medical persons add the sanction of their opinion, and consequently against one, to which the junior part of our profession may feel reluctant to oppose themselves—while the unhappy sufferers from hæmorrhoidal disease are willing to lay hold of any promised means of relief, and medical men have given their ineffectual aid both in a general and topical remedial way; surely it is neither good sense nor good practice to decry the only method, which offers a certain remedy for the most aggravated form of a malady, curable by no other means within the reach of our art, with either equal security or certainty." 121.

Our author's experience induces him to go further, even in cases where the hæmorrhoids seem to exercise a sanative power. In such cases, by adhering to the principles of the operation, we can, he thinks, extend a degree of relief to the patient which no other means are capable of affording. Mr. K. has not hesitated to use the knife, "in cases in which the discharge of hæmorrhoidal blood, from its long continuance, might be supposed to constitute almost a function belonging to the system." In these, he observes, we can with impunity remove much of the disease of the parts, and with them a great source of local irritation.

From some passages in the work reviewed, we are led to infer that there is much greater prejudice against excision of hæmorrhoidal tumours in Ireland than in this country. The operation is often performed here, both by the knife and by the ligature. Some serious accidents, however, have happened from the former mode, which have caused some dread of the operation. Yet, upon the whole, we think the knife may be the safer, as it certainly is the speedier and less painful mode of removing these troublesome and harrassing tumours.

